# Tri-Level Consistency–Diversity Calibration for Multi-View Representation Learning

**DOI:** 10.3390/e28050520

**Published:** 2026-05-04

**Authors:** Jinhui Hu, Lihong Qiao, Yucheng Shu

**Affiliations:** 1 School of Computer Science and Technology (National Exemplary Software School), Chongqing University of Posts and Telecommunications, Chongqing 400065, China; 2023212011@stu.cqupt.edu.cn (J.H.); shuyc@cqupt.edu.cn (Y.S.); 2 Chongqing Key Laboratory of Precision Diagnosis and Treatment for Kidney Diseases, No. 2 Chongwen Road, Nan’an District, Chongqing 400065, China

**Keywords:** multi-view representation learning, contrastive learning, consistency–diversity calibration, feature alignment

## Abstract

Robust representation learning from multi-source data necessitates the effective orchestration of complementary information while preserving semantic integrity. Existing methods primarily focus on class-level or instance-level alignment, neglecting fine-grained feature consistency and hierarchical collaborative mechanisms, which consequently limits representation precision. To address these issues, we propose the Tri-Level Consistency–Diversity Calibration (TCDC) method, a hierarchical framework designed to optimize information flow across feature, instance, and class levels. Specifically, at the feature level, TCDC imposes a variance–covariance constraint to align fine-grained features, thereby decorrelating dimensions. At the instance level, semantics-guided multi-objective graph learning is integrated with contrastive learning to adaptively calibrate global topology and capture high-order category correlations. Finally, a class-level attraction–repulsion constraint leverages category prototypes as global semantic anchors to promote intra-class aggregation and enhance inter-class separability. Extensive experiments on multiple public datasets demonstrate the effectiveness of TCDC.

## 1. Introduction

Multi-view learning (MVL) seeks to aggregate complementary information across diverse modalities, offering superior robustness and noise resilience compared to single-view paradigms [1,2,3,4,5]. With the rapid development of knowledge-driven fields such as intelligent video surveillance [6,7], cross-modal retrieval [8,9], and disease diagnosis [10,11], MVL has shown promising performance for a wide range of tasks, including video content analysis, object recognition, and deep semantic understanding. However, from an information-theoretic perspective, as data heterogeneity scales, effectively calibrating the balance between extracting shared mutual information and preserving view-specific information remains a fundamental challenge [12,13,14]. This imbalance fundamentally constrains the precision and scalability of current knowledge representations.

While subspace mapping, graph learning, and contrastive methods have advanced cross-view alignment [4,5,8,9,10,11,12,13,14,15,16], existing frameworks predominantly operate at the instance level or cluster level [17,18,19]. Consequently, the fine-grained semantic consistency at the feature level is frequently neglected. Furthermore, despite isolated attempts at multi-level alignment, a systematic mechanism capable of orchestrating information flow across a complete feature–instance–class hierarchy remains largely unexplored [17,20], limiting the model’s capacity to resolve complex semantic ambiguities.

To address these challenges, we propose the Tri-Level Consistency–Diversity Calibration (TCDC) method, a hierarchical framework designed to systematically regulate the information distribution across feature, instance, and class levels. Specifically, at the feature level, a Center–Variance–Covariance constraint explicitly decorrelates dimensions to reduce redundancy while maintaining a variance lower bound to prevent dimensional collapse. At the instance level, a multi-objective regularized graph learning module dynamically models high-order semantic topologies to calibrate feature distances, thereby unifying global structural priors with local contrastive alignment. At the class level, category prototypes serve as global semantic anchors to promote intra-class aggregation. In addition, a dynamic attraction–repulsion constraint is applied to expand class boundaries, effectively suppressing inter-class confusion and promoting class disentanglement. Extensive experiments on multiple public datasets demonstrate that TCDC outperforms state-of-the-art methods in multi-view representation learning tasks.

The main contributions of this work can be summarized as follows:

- We propose a progressive multi-view representation learning framework that systematically regularizes the embedding space across feature, instance, and class levels, thereby enhancing the accuracy and discriminability of the representations.
- At the feature level, we introduce a Center–Variance–Covariance constraint that minimizes off-diagonal covariance and enforces a variance lower bound to yield linearly decorrelated embeddings.
- At the instance level, we optimize a multi-objective graph to derive a semantic distance matrix, effectively embedding high-order topological structures into local contrastive alignment.
- At the class level, we couple contrastive semantic binding with a hard-negative penalty to simultaneously maximize intra-class aggregation and sharpen inter-class decision boundaries.

## 2. Related Work

### 2.1. Multi-View Contrastive Learning

Contrastive learning, which has recently emerged as a powerful approach [21,22], has demonstrated remarkable discriminative capability in multi-view representation learning tasks [23,24]. Its core mechanism operates by constructing positive and negative sample pairs to optimize a contrastive loss [15,16]. From an information-theoretic perspective, minimizing this contrastive loss is mathematically equivalent to maximizing a tractable lower bound on the mutual information between heterogeneous views.

Recent frameworks have introduced advanced regularization mechanisms to enhance representation quality. For instance, MCGC [25] employs graph filtering to suppress high-frequency noise, identifying a consensus structure via contrastive regularization. Addressing feature alignment, DealMVC [26] utilizes global and local contrastive calibration to optimize cross-view consistency, while Xu et al. [17] proposed a fusion-free, multi-level hierarchy to balance cross-view consistency with view-specific information. From an information-theoretic perspective, maximizing mutual information remains a core objective for capturing scene semantics as demonstrated by CMC [27]. Extending this, Lin et al. [28] minimized conditional entropy to facilitate missing-view recovery, and MMCLR [29] adapts these principles to multi-behavior recommendation systems, jointly optimizing interactions across multiple behaviors, multiple views, and behavior distinctions.

In the realm of unsupervised and semi-supervised learning, Li et al. [30] proposed Contrastive Clustering (CC), which implements end-to-end dual optimization of instance features and cluster assignments. To enhance discriminability, Wang et al. [31] designed a Cross-Level Discrimination (CLD) approach, integrating inter-instance similarity into contrastive learning between instances and local instance groups, thereby enhancing both generalization and robustness. Extending to semi-supervised settings, Li et al. [32] introduced CoMatch, a unified framework that leverages graph-based regularization and smoothness constraints to learn class probabilities and low-dimensional embeddings, resulting in improved representation and classification performance.

### 2.2. Graph-Based Multi-View Learning

Graph-based approaches in multi-view learning have emerged as a powerful paradigm for clustering, garnering substantial research interest [33,34,35,36]. By constructing similarity graphs to capture intrinsic data relationships, these approaches facilitate effective feature fusion and clustering [37,38,39].

Among these approaches, consensus graph learning is a representative paradigm. Li et al. [39] constructed essential similarity graphs in spectral space, employing weighted tensor nuclear norms to enforce high-order consistency and suppress noise. Similarly, Wang et al. [40] developed a framework that jointly optimizes view-specific and unified matrices. By adaptively weighting view contributions, it refines graph structures to directly generate robust clusters.

Another significant line of research explores parameter-free approaches. Wu et al. [41] leveraged two-layer diversity to mitigate inter-view inconsistencies, capturing feature and linkage discrepancies to derive consensus embeddings via kernel methods. Tang et al. [42] proposed Cross-view Graph Diffusion (CGD), utilizing iterative diffusion to capture manifold geometry and fuse complementary information. Furthermore, Li et al. [43] developed a scalable fusion strategy that integrates view-wise graphs via self-supervised weighting under connectivity constraints, achieving unification without hyperparameters.

Additionally, tensor-based representations have been explored to model relationships among multi-view data. In multi-view spectral clustering, Jia et al. [44] introduced a structured tensor low-rank norm that applies symmetric low-rank and structured sparse low-rank constraints to the frontal and horizontal slices of the tensor, respectively, capturing intra-view and inter-view relationships while enabling joint optimization for mutual refinement. Another approach was proposed by Wu et al. [45], who developed a Unified Graph and Low-Rank Tensor Learning (UGLTL) framework. This method simultaneously learns view-specific affinity matrices via projected graph learning and captures high-order correlations across views through low-rank approximation of the intrinsic tensor, jointly optimizing projection matrices, affinity matrices, and the intrinsic tensor to enhance multi-view clustering performance.

## 3. The Proposed Method

**Figure 1:**
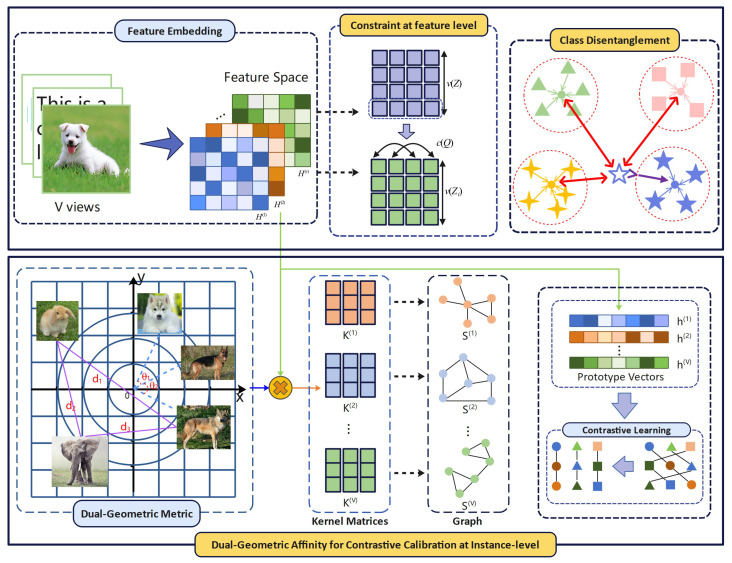
Overview of the proposed Tri-Level Consistency–Diversity Calibration (TCDC) framework. The method systematically orchestrates alignment across three hierarchies: (1) Feature Level: A Center–Variance–Covariance constraint ($L_{\mathrm{VIC}}$) decorrelates feature dimensions, thereby ensuring informative and diverse embeddings. (2) Instance Level: A multi-objective regularized graph learning module ($L_{\mathrm{IN}}$) models higher-order semantic correlations via prototype-guided affinity graphs, enhancing contrastive alignment. (3) Class Level: Semantic binding and class disentanglement ($L_{\mathrm{CAT}}$) leverage global prototypes as structural anchors to optimize intra-class compactness and expand inter-class decision boundaries.

In this section, we introduce the proposed Tri-Level Consistency–Diversity Calibration (TCDC) method, designed to systematically orchestrate consistency and diversity across feature, instance, and class hierarchies. As illustrated in Figure 1, the overall architecture consists of three collaboratively integrated modules: (1) Feature-Level Alignment: A Center–Variance–Covariance constraint ($L_{\mathrm{VIC}}$) linearly decorrelates feature dimensions and enforces a variance lower bound to prevent dimensional collapse, ensuring informative and diverse embeddings. (2) Instance-Level Calibration: A multi-objective regularized graph learning module ($L_{\mathrm{IN}}$) captures high-order semantic correlations via prototype-guided affinity graphs, topologically modulating feature distances for enhanced contrastive alignment. (3) Class-level Disentanglement: By leveraging global prototypes, the Semantic Binding and Class Disentanglement module ($L_{\mathrm{CAT}}$) promotes intra-class compactness while maximizing inter-class separability. The detailed formulations of these components are described in the following subsections.

### 3.1. Center–Variance–Covariance Constraint at Feature-Level

Multi-view embeddings often suffer inconsistency, redundancy, and mode collapse. We introduce a variance–covariance constraint, combining a covariance penalty and a hinge-based variance lower bound to jointly promote linear decorrelation and intra-class compactness.

Let $h_{i,j}^{\left(v\right)}\in R^{d}$ denote the latent representation extracted by the view-specific autoencoder for the *j*-th sample of class *i* under view *v*. We then compute mean-centered representations $g_{i,j}^{\left(v\right)}$ that form the class-specific and global feature matrices $G_{i}^{\left(v\right)}$ and $G^{\left(v\right)}$.

To penalize inter-dimensional dependencies, we introduce a local covariance constraint. Minimizing the squared off-diagonal covariance elements serves as a tractable regularizer that encourages linear decorrelation among feature dimensions. This explicitly prevents different dimensions from encoding redundant information, thereby maximizing the utilization of the embedding space:

(1) $$c\left(Q\right)=\frac{1}{V}\sum_{v=1}^{V}\frac{1}{C}\sum_{i=1}^{C}\frac{1}{d}\sum_{j\neq k}{\left({\left[Q_{i}^{\left(v\right)}\right]}_{j,k}\right)}^{2},$$

where $Q_{i}^{\left(v\right)}=\frac{1}{n_{i}}{\left(G_{i}^{\left(v\right)}\right)}^{⊤}G_{i}^{\left(v\right)}$.

Unconstrained variance minimization often induces dimensional collapse. To prevent it, we introduce a hinge-based variance regularizer that enforces a lower bound on the standard deviation for each feature dimension within intra-class:

(2) $$v\left(G_{i}\right)=\frac{1}{V}\sum_{v=1}^{V}\frac{1}{C}\sum_{i=1}^{C}\frac{1}{d}\sum_{j=1}^{d}\frac{\mathrm{ReLU}\left(1-\sigma_{i,j}^{\left(v\right)}\right)}{2},$$

where $\sigma_{i,j}^{\left(v\right)}=\sqrt{\mathrm{Var}\;\left({\left[G_{i}^{\left(v\right)}\right]}_{:,j}\right)+\epsilon }$. An analogous constraint v(G) applies to the global distribution to maintain overall spatial diversity.

Integrating these components, the feature-level alignment is formulated as

(3) $$L_{\mathrm{VIC}}=\alpha_{1}\frac{v(G_{i})}{v(G)+\epsilon }+c\left(Q\right).$$

Minimizing the ratio $v\left(G_{i}\right)/v\left(G\right)$ balances intra-class compactness and global diversity. The hyperparameter $\alpha_{1}$ controls the trade-off between this variance ratio and the decorrelation term c(Q).

### 3.2. Multi-Objective Regularized Graph Learning at Instance-Level

With the proliferation of multi-source heterogeneous data, discovering shared patterns across data sources has become essential. While instance-level contrastive learning enhances local discriminability by maximizing mutual information between congruent views [23,27], it primarily focuses on sample-wise alignment and fails to explicitly model global topological structures.

To address this, graph-based methods have been widely adopted. However, existing approaches exhibit limitations in capturing high-order semantic correlations. For instance, while some research recognizes the inadequacy of Euclidean metrics and dynamically learn affinity graphs using alternative measures [46], they primarily focus on sample-level pairwise relationships without explicitly leveraging global macroscopic structures. Other approaches, such as consensus graph learning [41,47] or cross-view graph diffusion [42], primarily focus on propagating local similarities or learning embeddings from predefined affinity matrices. They often lack explicit constraints to capture high-order, category-level semantic correlations during the graph learning process.

To overcome these limitations, we propose a multi-objective regularized graph learning model at the instance level. Rather than relying solely on sample-level affinities, we dynamically optimize the graph structure guided by global semantics. We first initialize a structural prior kernel $K^{\left(v\right)}$ using a Gaussian–cosine hybrid metric to capture both spatial proximity and directional geometry. Using category prototypes ${\bar{H}}^{\left(v\right)}$ as global semantic anchors, we formulate the following joint optimization objective to learn the optimal affinity matrix $Z^{\left(v\right)}$:

(4) $$\begin{array}{cc} \min_{Z^{\left(v\right)}}\{ & \frac{1}{2}\sum_{i,j,k}k_{i,j}^{\left(v\right)}{\left(z_{i,k}^{\left(v\right)}-z_{j,k}^{\left(v\right)}\right)}^{2}+\mu_{1}{∥Z^{\left(v\right)}-K^{\left(v\right)}∥}_{F}^{2}\\ \; & +\mu_{2}∥Z^{\left(v\right)}{-I∥}_{F}^{2}+\mu_{3}{∥Z^{\left(v\right)}∥}_{F}^{2}+\mu_{4}{∥{\bar{H}}^{\left(v\right)}-Z^{\left(v\right)}{\bar{H}}^{\left(v\right)}∥}_{F}^{2}\},\\ \; & \;\mathrm{subject}\;\mathrm{to}\;Z^{\left(v\right)}1_{C}=1_{C}. \end{array}$$

Here, the first two terms ensure manifold smoothness and topological fidelity to the prior kernel. The subsequent terms impose identity constraints, sparsity, and low-rank self-representation via category prototypes ${\bar{H}}^{\left(v\right)}$. This ensures that the dynamically learned graph $Z^{\left(v\right)}$ captures complex, higher-order intrinsic relationships rather than merely local neighborhood similarities.

After solving for $Z^{\left(v\right)}$ via ADMM and averaging across views to obtain the global affinity $\bar{Z}$, we invert it into a semantic distance matrix S. To seamlessly integrate this global topology with local alignment, we use S to modulate the feature distances D. This yields two weighted distance metrics: intra-class compactness ($d_{\mathrm{same}}$) and inter-class separability ($d_{\mathrm{diff}}$):

(5) $$d_{\mathrm{same}}=\frac{\sum S_{i,k}D_{\left(i,j),(k,\ell \right)}Y_{\left(i,j),(k,\ell \right)}}{\sum Y_{\left(i,j),(k,\ell \right)}+\epsilon },\;d_{\mathrm{diff}}=\frac{\sum S_{i,k}D_{\left(i,j),(k,\ell \right)}\left(1-Y_{\left(i,j),(k,\ell \right)}\right)}{\sum (1-Y_{\left(i,j),(k,\ell \right)})+\epsilon }$$

where Y is the class indicator matrix. The final instance-level objective maximizes mutual information via contrastive learning ($L_{\mathrm{ins}}$) while simultaneously preserving the underlying category relationships among features through the dynamically learned topological calibration:

(6) $$L_{\mathrm{IN}}=\alpha_{2}L_{\mathrm{ins}}+\frac{d_{\mathrm{same}}}{d_{\mathrm{diff}}+\epsilon }$$

where $\alpha_{2}$ is a balancing hyperparameter that regulates the relative contribution of the mutual information maximization and the global topological calibration.

### 3.3. Semantic Binding and Class Disentanglement at Class-Level

We establish global class centers $\mu_{i}$ by aggregating features across all views and samples within the same category. Using these centers as global anchors, we enforce semantic binding by maximizing the mutual information between each sample and its corresponding class center via contrastive learning:

(7) $$L_{\mathrm{cls}}=\frac{1}{C}\sum_{i=1}^{C}\frac{1}{V}\sum_{v=1}^{V}\frac{1}{n_{i}}\sum_{j=1}^{n_{i}}-\log\left(\frac{\exp\left(\langle h_{i,j}^{\left(v\right)},\mu_{i}\rangle /\tau_{2}\right)}{\sum_{k\neq i}\exp\left(\langle h_{i,j}^{\left(v\right)},\mu_{k}\rangle /\tau_{2}\right)+\epsilon }\right),$$

where $\tau_{2}$ is the temperature parameter. To further strengthen class boundaries, we couple this semantic binding with a hard-negative disentanglement penalty, forming the joint class-level objective ($L_{\mathrm{CAT}}$):

(8) $$L_{\mathrm{CAT}}=\alpha_{3}L_{\mathrm{cls}}+\frac{1}{C}\sum_{i=1}^{C}\frac{1}{n_{i}}\sum_{j=1}^{n_{i}}\frac{∥{\hat{h}}_{i,j}-\mu_{i}{∥}_{2}^{2}}{\min_{k\neq i}{∥{\hat{h}}_{i,j}-\mu_{k}∥}_{2}^{2}+\epsilon },$$

where the second term actively minimizes the sample-to-center distance while explicitly maximizing the distance to the nearest non-target class center.

Finally, incorporating the standard cross-entropy loss ($L_{\mathrm{class}}$) for classification, the overall optimization objective integrates the reconstruction loss ($L_{\mathrm{recon}}$) with feature-level ($L_{\mathrm{VIC}}$), instance-level ($L_{\mathrm{IN}}$), and class-level ($L_{\mathrm{CAT}}$) constraints:

(9) $$L_{\mathrm{total}}=L_{\mathrm{recon}}+L_{\mathrm{class}}+\alpha L_{\mathrm{VIC}}+\beta L_{\mathrm{IN}}+\gamma L_{\mathrm{CAT}},$$

where α,β, and γ are hyperparameters balancing the respective components.

### 3.4. Optimization

In this section, we optimize the affinity matrix $Z^{\left(v\right)}$ using the Alternating Direction Method of Multipliers (ADMM). We introduce an auxiliary variable $J^{\left(v\right)}$ to decouple the row-sum constraint, reformulating the problem as

(10) $$\begin{array}{cc} \min_{Z^{\left(v\right)},J^{\left(v\right)}}\{ & \frac{1}{2}\sum_{i,j,k}k_{i,j}^{\left(v\right)}{\left(z_{i,k}^{\left(v\right)}-z_{j,k}^{\left(v\right)}\right)}^{2}+\mu_{1}{∥Z^{\left(v\right)}-K^{\left(v\right)}∥}_{F}^{2}\\ \; & +\mu_{2}∥Z^{\left(v\right)}{-I∥}_{F}^{2}+\mu_{3}{∥Z^{\left(v\right)}∥}_{F}^{2}+\mu_{4}{∥{\bar{H}}^{\left(v\right)}-J^{\left(v\right)}{\bar{H}}^{\left(v\right)}∥}_{F}^{2}\},\\ \; & \;s.t.\;Z^{\left(v\right)}=J^{\left(v\right)},\;J^{\left(v\right)}1_{C}=1_{C} \end{array}$$

Let $M^{\left(v\right)}=L^{\left(v\right)}+\left(\mu_{1}+\mu_{2}+\mu_{3}\right)I$ and $Q^{\left(v\right)}=\mu_{1}K^{\left(v\right)}+\mu_{2}I$, where $L^{\left(v\right)}$ is the Laplacian matrix. By constructing the augmented Lagrangian with a scaled dual variable $U^{\left(v\right)}\in R^{C\times C}$ and penalty ρ, the alternating update steps are directly given as follows:

- Step 1: Update $Z^{\left(v\right)}$

(11) $$Z^{\left(v)(t+1\right)}={\left(2M^{\left(v\right)}+\rho I\right)}^{-1}\left(2Q^{\left(v\right)}+\rho \left(J^{\left(v)(t\right)}-U^{\left(v)(t\right)}\right)\right)$$

- Step 2: Update $J^{\left(v\right)}$

Defining $A^{\left(v\right)}={\bar{H}}^{\left(v\right)}{\left({\bar{H}}^{\left(v\right)}\right)}^{⊤}$, $B^{\left(v\right)}=Z^{\left(v)(t+1\right)}+U^{\left(v)(t\right)}$, $R^{\left(v\right)}=2\mu_{4}A^{\left(v\right)}+\rho I$, and $T^{\left(v\right)}=2\mu_{4}A^{\left(v\right)}+\rho B^{\left(v\right)}$, the closed-form solution enforcing $J^{\left(v\right)}1_{C}=1_{C}$ is

(12) $$J^{\left(v)(t+1\right)}=\left(T^{\left(v\right)}-\lambda 1_{C}^{⊤}\right){\left(R^{\left(v\right)}\right)}^{-1}$$

where the Lagrange multiplier vector is given by $\lambda =\frac{T^{\left(v\right)}{\left(R^{\left(v\right)}\right)}^{-1}1_{C}-1_{C}}{1_{C}^{⊤}{\left(R^{\left(v\right)}\right)}^{-1}1_{C}}$.

- Step 3: Update $U^{\left(v\right)}$

(13) $$U^{\left(v)(t+1\right)}=U^{\left(v)(t\right)}+Z^{\left(v)(t+1\right)}-J^{\left(v)(t+1\right)}$$

Repeat the above steps until convergence.

## 4. Experiments

To rigorously evaluate the effectiveness and robustness of the proposed method, we conduct comprehensive experiments on six standard multi-view benchmark datasets: HandWritten (https://archive.ics.uci.edu/ml/datasets/Multiple+Features, accessed on 15 January 2026), Scene15 (https://doi.org/10.6084/m9.figshare.7007177.v1, accessed on 15 January 2026), PIE (https://www.cs.cmu.edu/afs/cs/project/PIE/MultiPie/Multi-Pie/Home.html, accessed on 15 January 2026), CCV (http://www.ee.columbia.edu/ln/dvmm/CCV, accessed on 15 January 2026), 100Leaves (https://github.com/ChuanbinZhang/Multi-view-datasets, accessed on 15 January 2026), and Hdigit (https://github.com/dejavu-git/datasets/blob/main/Hdigit.mat, accessed on 15 January 2026). These datasets cover a wide range of domains, including scene classification, digit recognition, face analysis, and plant species identification. A concise statistical summary of these datasets, including the number of instances (*N*), classes (*C*), views (*V*), and feature dimensions, is provided in Table 1. Detailed descriptions of each dataset are given as follows:

Scene15: This dataset serves as a standard benchmark for scene recognition, comprising 4485 images distributed across 15 distinct scene categories from both indoor and outdoor environments. Each image is represented by three types of visual features: GIST, PHOG, and LBP.

HandWritten: This is a classic dataset for digit recognition, encompassing 2000 samples of handwritten digits (0–9) organized into 10 classes, with 200 balanced instances per class. Each sample is characterized by six distinct feature representations, providing a rich multi-view context.

PIE: This dataset contains 680 facial images corresponding to 68 distinct subjects. Each image is described by three feature views: intensity, LBP, and Gabor.

CCV: This is a large-scale video dataset consisting of 6773 samples collected from YouTube, spanning 20 semantic categories. Each video is characterized by three distinct audio-visual feature views.

100Leaves: This dataset is designed for plant species identification, consisting of 1600 samples from 100 distinct plant classes. Each sample is described by three geometric and textural feature views: shape descriptors, fine-scale margins, and texture histograms.

Hdigit: This is a large-scale handwritten digit recognition dataset containing 10,000 samples across 10 digit classes. This dataset tests the scalability of the model and is represented by two distinct feature views.

### 4.1. Implementation Details

The proposed multi-view learning model is implemented using the PyTorch (version 2.7) framework. The encoder, $f_{v}^{\mathrm{enc}}:R^{d_{v}}\to R^{d}$, and the decoder, $f_{v}^{\mathrm{dec}}:R^{d}\to R^{d_{v}}$, are constructed as fully connected networks (MLPs). The layer dimensions for the encoder are set as $\left\{d_{v},256,128,128\right\}$, and the decoder layer dimensions are $\left\{128,128,256,d_{v}\right\}$, where $d_{v}$ denotes the original feature dimension of the *v*-th view. The ReLU non-linear activation function is applied after every hidden layer. The model is optimized using the Adam optimizer with an initial learning rate of $5\times 10^{-4}$ and a weight decay of $10^{-4}$. We set the training batch size to 128 and train the model for 200 epochs. To dynamically adjust the learning rate, we utilize the StepLR scheduler, which decays the learning rate by a factor of 0.5 every 50 epochs. All experiments are conducted on a server equipped with an NVIDIA GeForce RTX 3090 GPU (24 GB memory).

### 4.2. Comparison with State-of-the-Art Methods

To demonstrate the superior efficacy and competitiveness of the proposed model, we conduct comprehensive comparisons against seven state-of-the-art multi-view learning methods: mmdynamics [48], ETMC [49], UMDL [50], PDMF [51], MAMC [52], MV-HFMD [53], and RCML [54]. For fairness and rigorous evaluation, we adopt a standard 80/20 data splitting strategy, allocating 80% of the instances for training and the remaining 20% for testing. To ensure the reliability and statistical significance of the results, the splitting process is repeated ten times, and the average performance is reported. We utilize accuracy, precision, recall, and Macro-F1 score as evaluation metrics.

As shown in Table 2, our method consistently outperforms all baselines. Notably, on challenging datasets such as Scene15 and CCV that require fine-grained semantic separation, our model surpasses the second-best baseline MAMC by margins of 4.6% and 5.7%, respectively. While methods like ETMC and UMDL struggle with complex class boundaries in these scenarios, our approach resolves this by explicitly strengthening discriminative disentanglement. Furthermore, our model yields consistent improvements even on highly saturated datasets like HandWritten and 100Leaves, where baseline accuracies already exceed 98%. In summary, our hierarchical framework achieves superior robustness and precision by systematically orchestrating information across the feature, instance, and class levels.

**Figure 2:**
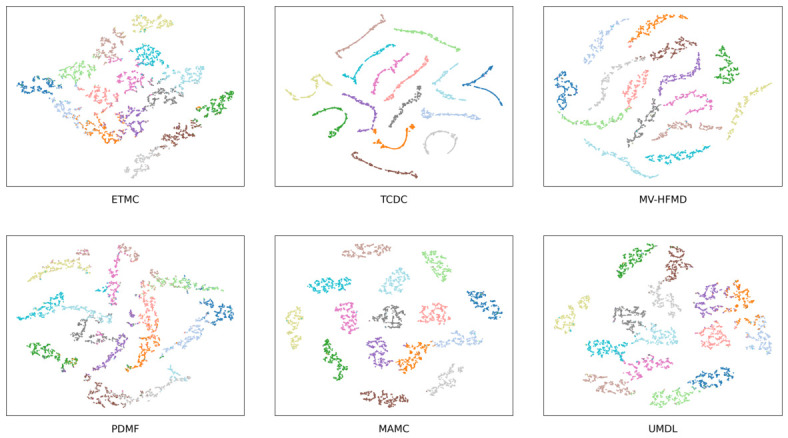
t-SNE visualizations on the Scene15 dataset. Colors denote ground-truth categories. Results of different methods (ETMC, UMDL, PDMF, MV-HFMD, MAMC, and TCDC) are shown.

To intuitively demonstrate the effectiveness of TCDC in learning discriminative multi-view representations, we employ t-SNE to project high-dimensional embeddings into a 2D space. Figure 2 presents the visualization results of TCDC and representative baselines on the Scene15 dataset.

Furthermore, to assess the statistical significance of the observed performance gains, we perform paired *t*-tests at a significance level of 0.05. Specifically, accuracy scores obtained from 10 independent runs are compared against three top baselines (RCML, MAMC, and MV-HFMD). As reported in Table 3, TCDC achieves statistically significant improvements (p<0.001) in 11 out of 12 experimental settings. The only exception is observed on the Hdigit dataset when compared with MAMC (p=0.23). These results provide strong evidence for the robustness and effectiveness of our method. Finally, to evaluate computational efficiency, we compare TCDC with PDMF, UMDL, and ETMC under identical settings on a single RTX 3090 GPU. The detailed results are summarized in Table 4.

### 4.3. Ablation Study

To systematically evaluate the effectiveness of the proposed Tri-Level Consistency–Diversity Calibration (TCDC) framework and its underlying mechanisms, we conduct comprehensive ablation studies on the highly challenging Scene15 and CCV datasets.

We decoupled the internal sub-constraints of the three core modules ($L_{VIC}$, $L_{IN}$, and $L_{CAT}$) to verify the optimality of their designs. The quantitative results are presented in Table 5, Table 6 and Table 7. The consistent performance degradation observed when deploying isolated sub-constraints quantitatively confirms the necessity of our design. Specifically, the joint optimization of feature-level Variance–Covariance Constraint, instance-level topological calibration, and class-level boundary disentanglement is strictly indispensable for achieving optimal discriminability.

To validate the structural superiority of the Multi-Objective Regularized Graph (MORG), we conducted comparative experiments against traditional graph methods. As detailed in Table 8, replacing MORG with a Fully Connected Graph (FCG) or k-Nearest Neighbor (k-NN) graph results in a significant drop in performance. Unlike traditional methods, which propagate noise due to uncalibrated metrics, MORG utilizes structural constraints to align embeddings with the intrinsic semantic topology.

Furthermore, to verify the synergistic effects among the three hierarchical modules, we conduct an incremental ablation study on the Scene15 and CCV datasets as shown in Table 9. Starting from the feature-level constraint, adding the instance-level alignment improves classification accuracy, and further incorporating the class-level constraint achieves the highest performance. These results confirm that the hierarchical modules progressively enhance each other.

To further validate the necessity of the proposed tri-level synergistic mechanism, we conduct an ablation study as shown in Table 10. Removing any individual module ($L_{VIC}$, $L_{IN}$, or $L_{CAT}$) consistently degrades classification performance. Optimal performance is achieved only through joint optimization, confirming that integrating structural constraints across all three hierarchies is indispensable for robust multi-view representation learning.

### 4.4. Convergence Analysis

**Figure 3:**
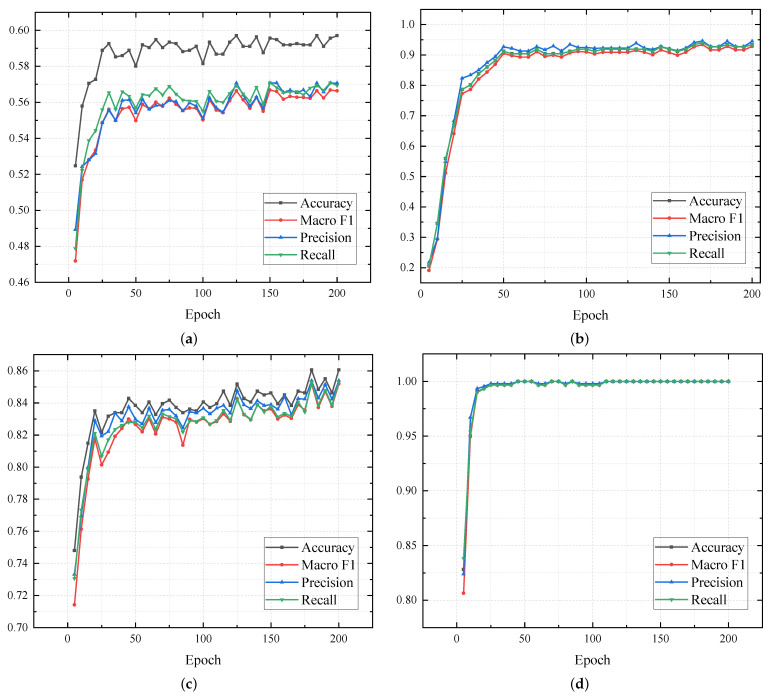
Convergence trajectories of accuracy, Macro-F1, precision, and recall for TCDC on four datasets. (**a**) CCV, (**b**) PIE, (**c**) Scene15, (**d**) 100Leaves.

To evaluate the numerical stability and optimization efficiency of our framework, we analyze the training trajectories across four representative datasets: Scene15, CCV, PIE, and 100Leaves. Figure 3 illustrates the trajectories of evaluation metrics over 200 epochs. As observed, the proposed method exhibits a rapid and robust convergence pattern across all scenarios. Specifically, on the 100Leaves dataset, performance metrics saturate and plateau within approximately 25 epochs. For more challenging datasets such as CCV and Scene15, the model attains a steady state after roughly 50 epochs despite minor initial fluctuations. The high synchronization among all evaluation metrics validates that the hierarchical constraints ($L_{\mathrm{VIC}}$, $L_{\mathrm{IN}}$, and $L_{\mathrm{CAT}}$) effectively cooperate without objective conflicts, ensuring reliable training in complex multi-view spaces.

### 4.5. Parameter Analysis

To evaluate the robustness and optimal configuration of our framework, we conduct sensitivity tests on three groups of hyperparameters: the temperature parameters $\tau_{1}$ and $\tau_{2}$, the intra-module weighting coefficients $\alpha_{1},\;\alpha_{2}$, and $\alpha_{3}$, and the inter-module weighting coefficients α,β, and γ.

**Figure 4:**
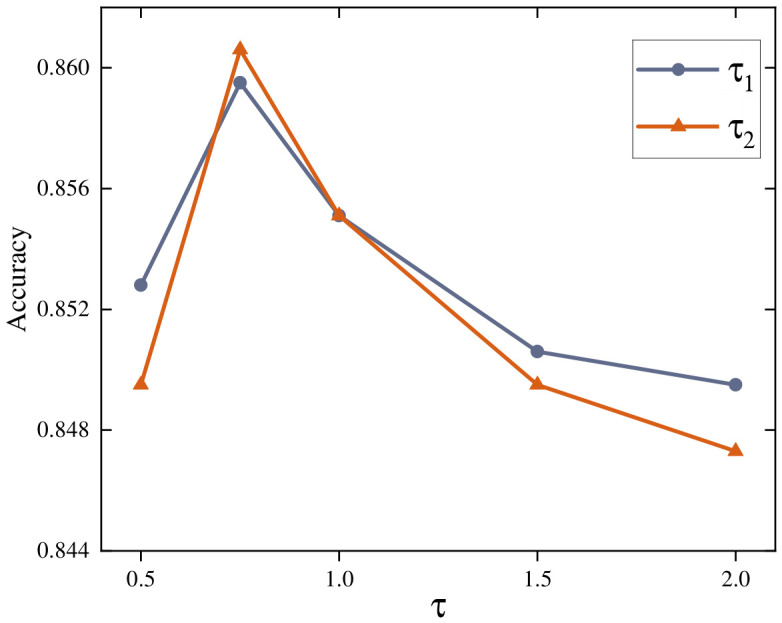
Sensitivity analysis of temperature parameters $\tau_{1}$ and $\tau_{2}$ on the Scene15 dataset.

Temperature parameters $\tau_{1}$ and $\tau_{2}$ regulate the distribution sharpness for instance-level and class-level alignments. We conduct sensitivity tests on the Scene15 dataset by varying both parameters within {0.5,1.0,1.5,2.0}. As shown in Figure 4, the accuracy follows a consistent bell-shaped trend, peaking near 0.75. Lower values such as 0.5 induce overly sharp distributions that may over-penalize hard negatives and amplify noise. Conversely, increasing the temperature beyond 1.0 leads to excessive smoothing, which blurs the semantic boundaries between categories and degrades discriminability. Consequently, we empirically set both $\tau_{1}$ and $\tau_{2}$ to 0.75 to achieve an optimal balance between alignment stiffness and distribution smoothness.

**Figure 5:**
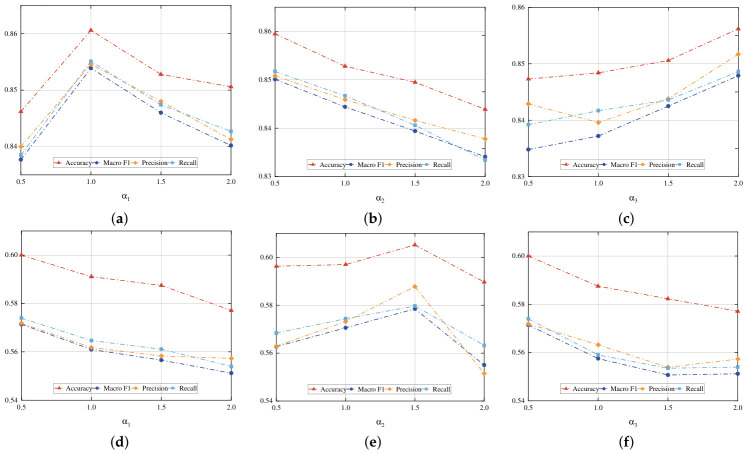
Sensitivity analysis of intra-module weights $\alpha_{1},\alpha_{2}$, and $\alpha_{3}$. The top row (**a**–**c**) shows results on the Scene15 dataset, while the bottom row (**d**–**f**) represents the CCV dataset. (**a**) Scene15: $\alpha_{1}$, (**b**) Scene15: $\alpha_{2}$, (**c**) Scene15: $\alpha_{3}$, (**d**) CCV: $\alpha_{1}$, (**e**) CCV: $\alpha_{2}$, (**f**) CCV: $\alpha_{3}$.

We evaluate the internal balance of hierarchical modules via sensitivity analysis on $\alpha_{1},\;\alpha_{2}$, and $\alpha_{3}$. These parameters are varied across the set {0.5,1.0,1.5,2.0} on the Scene15 and CCV datasets. As illustrated in Figure 5, the optimal values for these parameters vary significantly across these datasets. On Scene15, the Accuracy peaks at $\alpha_{1}=1.0$, while it exhibits a continuous decline on CCV, indicating that Scene15 requires a balanced variance–covariance constraint while CCV favors a smaller $\alpha_{1}$. For $\alpha_{2}$, the performance on Scene15 decreases as the value increases, whereas CCV shows a distinct bell-shaped curve peaking at 1.5, suggesting that the complex semantic structure of CCV necessitates a stronger emphasis on contrastive alignment. Turning to $\alpha_{3}$, performance improves with higher values on Scene15 but drops sharply on CCV, reflecting the distinct requirements for semantic binding and distance constraints across these two datasets.

**Figure 6:**
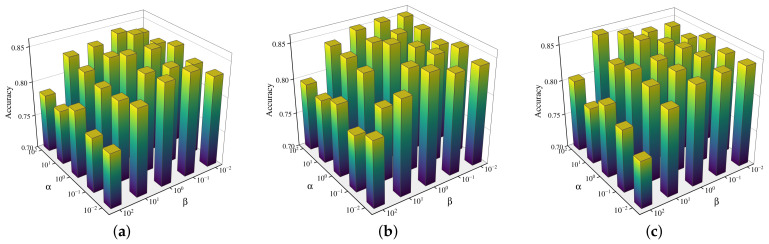
Sensitivity analysis of inter-module weights α,β, and γ on the Scene15 dataset. The 3D bar charts illustrate the accuracy variation across a grid search of α and β under different γ scales. (**a**) γ=0.1, (**b**) γ=1, (**c**) γ=10.

Finally, we investigate the interplay between the hierarchical modules by conducting a grid search on the inter-module weights α,β, and γ. With γ fixed at {0.1,1,10}, the parameters α and β are varied across the logarithmic scale $\left\{10^{-2},10^{-1},10^{0},10^{1},10^{2}\right\}$ on the Scene15 dataset. As illustrated in the 3D bar charts in Figure 6, the accuracy remains relatively stable across a wide range of parameter combinations, demonstrating the robustness of the joint optimization objective. Optimal performance is generally achieved when α and β are situated in the central region (around $10^{0}$ to $10^{1}$), while γ=1 provides a balanced contribution for class-level constraints. The results confirm that the integration of feature-level, instance-level, and class-level modules effectively enhances the discriminative power of multi-view representations.

## 5. Conclusions

In this paper, we propose TCDC, a hierarchical framework that systematically orchestrates consistency and diversity across feature, instance, and class levels. By synergizing variance–covariance decorrelation, multi-objective regularized graph learning, and semantic-binding disentanglement, TCDC effectively balances global topological fidelity with discriminability. Extensive experimental evidence demonstrates that explicitly managing the hierarchical information flow not only yields superior representation performance but also guarantees robust generalization across complex semantic distributions. Future work will extend this information-theoretic framework to address the challenge of missing modalities in incomplete multi-view learning and explore its theoretical scalability within the context of the Information Bottleneck principle.

## Figures and Tables

**Table 1:** Summary of the characteristics of the employed multi-view datasets.

Dataset	Instances	Classes	Views	View Dimensions
Scene15	4485	15	3	20/59/40
HandWritten	2000	10	6	240/76/216/47/64/6
PIE	680	68	3	484/256/279
CCV	6773	20	3	20/20/20
100Leaves	1600	100	3	60/60/60
Hdigit	10,000	10	2	784/256

**Table 2:** Classification performance (mean ± std, %) of different multi-view learning methods on six benchmark datasets. The best results are highlighted in bold.

Datasets	Metric	Baseline Models							Ours
		RCML	MMDynamics	MAMC	PDMF	UMDL	ETMC	MV-HFMD	
Scene15	Acc	70.0 ± 1.0	62.0 ± 2.2	81.5 ± 0.7	67.8 ± 1.1	63.3 ± 0.4	66.5 ± 1.8	80.7 ± 1.2	**86.1 ± 0.7**
	Precision	70.6 ± 1.3	57.3 ± 2.8	81.0 ± 0.6	65.8 ± 2.8	61.2 ± 0.7	66.6 ± 2.5	80.6 ± 1.6	**85.4 ± 0.6**
	Recall	68.9 ± 1.3	60.1 ± 2.2	80.8 ± 0.7	65.9 ± 1.1	62.7 ± 0.5	65.3 ± 1.6	80.3 ± 1.3	**85.3 ± 0.7**
	Macro-F1	67.1 ± 1.1	57.6 ± 2.5	80.5 ± 0.7	63.0 ± 1.4	61.1 ± 0.7	62.8 ± 1.8	80.0 ± 1.4	**85.2 ± 0.7**
HandWritten	Acc	97.1 ± 0.9	98.5 ± 0.5	98.9 ± 0.4	98.2 ± 0.7	98.5 ± 0.5	96.8 ± 0.7	98.2 ± 0.5	**99.0 ± 0.3**
	Precision	97.1 ± 0.9	98.5 ± 0.5	98.9 ± 0.4	98.3 ± 0.7	97.8 ± 0.5	96.8 ± 0.7	98.2 ± 0.5	**99.1 ± 0.3**
	Recall	97.2 ± 0.9	98.5 ± 0.5	98.9 ± 0.4	98.3 ± 0.7	97.7 ± 0.5	96.9 ± 0.7	98.2 ± 0.6	**99.0 ± 0.2**
	Macro-F1	97.1 ± 0.9	98.5 ± 0.5	98.9 ± 0.4	98.2 ± 0.7	97.7 ± 0.5	96.8 ± 0.7	98.1 ± 0.5	**99.0 ± 0.2**
Hdigit	Acc	98.3 ± 0.3	99.6 ± 0.1	**99.8 ± 0.1**	99.4 ± 0.2	98.0 ± 0.2	98.8 ± 2.1	84.1 ± 2.0	**99.8 ± 0.1**
	Precision	98.3 ± 0.3	99.6 ± 0.1	**99.8 ± 0.1**	99.4 ± 0.2	98.0 ± 0.2	98.4 ± 0.2	88.9 ± 1.7	**99.8 ± 0.1**
	Recall	98.3 ± 0.3	99.6 ± 0.1	**99.8 ± 0.1**	99.4 ± 0.2	98.0 ± 0.2	98.4 ± 0.2	87.5 ± 2.0	**99.8 ± 0.1**
	Macro-F1	98.3 ± 0.3	99.6 ± 0.1	**99.8 ± 0.1**	99.4 ± 0.2	98.0 ± 0.2	98.4 ± 0.2	86.5 ± 2.3	**99.8 ± 0.1**
CCV	Acc	42.4 ± 1.7	29.6 ± 1.1	54.0 ± 1.1	50.5 ± 1.4	36.2 ± 1.5	42.6 ± 1.4	53.4 ± 0.9	**59.7 ± 0.1**
	Precision	42.3 ± 2.2	22.8 ± 2.8	53.0 ± 1.3	48.5 ± 1.3	35.5 ± 1.3	41.4 ± 2.6	51.1 ± 1.1	**57.0 ± 0.7**
	Recall	37.0 ± 1.4	23.2 ± 1.0	50.6 ± 1.0	45.4 ± 1.3	33.7 ± 1.4	37.1 ± 1.4	50.5 ± 0.8	**56.9 ± 0.3**
	Macro-F1	35.9 ± 1.5	20.1 ± 1.1	51.0 ± 0.9	45.1 ± 1.5	33.1 ± 1.4	36.0 ± 1.6	50.4 ± 0.9	**56.6 ± 0.4**
PIE	Acc	91.8 ± 2.9	72.5 ± 3.7	93.1 ± 1.7	88.7 ± 3.0	71.1 ± 3.3	90.5 ± 2.3	86.8 ± 2.4	**94.1 ± 1.7**
	Precision	91.1 ± 3.4	70.6 ± 4.0	92.8 ± 2.1	87.6 ± 3.8	74.6 ± 3.9	88.0 ± 3.8	87.7 ± 2.7	**94.6 ± 2.0**
	Recall	91.0 ± 3.5	70.6 ± 3.7	93.6 ± 1.8	87.3 ± 4.9	71.1 ± 3.3	89.6 ± 3.6	88.9 ± 2.4	**94.1 ± 1.6**
	Macro-F1	89.8 ± 3.9	67.4 ± 3.8	92.1 ± 2.0	85.7 ± 4.5	69.3 ± 3.5	87.4 ± 3.8	83.8 ± 6.1	**93.4 ± 1.3**
100Leaves	Acc	88.6 ± 1.5	93.5 ± 1.5	98.5 ± 1.1	97.7 ± 0.7	98.4 ± 0.8	90.8 ± 2.1	98.3 ± 0.2	**100.0 ± 0.0**
	Precision	88.7 ± 2.0	93.6 ± 1.2	98.5 ± 1.0	97.7 ± 0.9	98.7 ± 0.8	90.5 ± 2.5	98.4 ± 0.0	**100.0 ± 0.0**
	Recall	89.4 ± 1.4	93.9 ± 1.1	99.0 ± 0.7	98.0 ± 1.0	98.4 ± 0.9	91.3 ± 2.4	98.9 ± 0.2	**100.0 ± 0.0**
	Macro-F1	87.0 ± 1.7	92.7 ± 1.3	98.5 ± 1.0	97.5 ± 1.0	98.3 ± 0.9	89.3 ± 2.7	98.4 ± 0.1	**100.0 ± 0.0**

**Table 3:** Paired *t*-test results (*p*-values) for accuracy comparisons between TCDC and baseline methods.

Datasets	RCML	MAMC	MV-HFMD
Scene15	$1.74\times 10^{-12}$	$2.38\times 10^{-9}$	$7.26\times 10^{-8}$
Handwritten	$6.81\times 10^{-7}$	$8.52\times 10^{-4}$	$5.29\times 10^{-5}$
Hdigit	$1.13\times 10^{-9}$	0.23	$1.51\times 10^{-11}$
PIE	$1.87\times 10^{-4}$	$1.59\times 10^{-8}$	$1.75\times 10^{-6}$

**Table 4:** Comparison of computational efficiency (memory, train time, and inference time) among different methods on a single RTX 3090 GPU.

Method	Memory (GB)	Train Time (h)	Inference Time (ms)
PDMF	1.2	0.69	16.8
UMDL	5.1	4.59	42.4
ETMC	2.1	1.47	20.5
Ours (TCDC)	2.6	2.41	24.6

**Table 5:** Ablation study of the fine-grained components at the feature level ($L_{VIC}$) on the Scene15 and CCV datasets. $L_{cov}$ and $L_{var}$ denote the covariance constraint and variance constraint. The best results are highlighted in bold.

Datasets	$L_{\mathrm{cov}}$	$L_{\mathrm{var}}$	Acc (%)	Precision (%)	Recall (%)	Macro-F1 (%)
Scene15			84.6	84.0	83.3	83.2
	✓		85.7	84.7	84.9	85.0
		✓	84.8	84.4	84.1	84.0
	✓	✓	**86.1**	**85.4**	**85.3**	**85.2**
CCV			58.7	56.0	56.3	55.8
	✓		59.5	56.8	57.0	56.4
		✓	58.9	56.2	56.2	55.9
	✓	✓	**59.7**	**57.0**	**56.9**	**56.6**

**Table 6:** Ablation study of the fine-grained components at the instance level ($L_{IN}$) on the Scene15 and CCV datasets. $L_{ins}$ and $L_{graph}$ denote the contrastive learning loss and multi-objective regularized graph constraint. The best results are highlighted in bold.

Datasets	$L_{\mathrm{ins}}$	$L_{\mathrm{graph}}$	Acc (%)	Precision (%)	Recall (%)	Macro-F1 (%)
Scene15			84.0	83.8	82.9	82.9
	✓		85.0	84.3	83.9	83.9
		✓	85.5	85.1	84.8	84.7
	✓	✓	**86.1**	**85.4**	**85.3**	**85.2**
CCV			57.5	55.2	55.3	55.0
	✓		58.6	55.6	55.9	55.5
		✓	59.2	56.0	56.4	55.8
	✓	✓	**59.7**	**57.0**	**56.9**	**56.6**

**Table 7:** Ablation study of the fine-grained components at the class level ($L_{CAT}$) on the Scene15 and CCV datasets. $L_{cls}$ and $L_{cd}$ denote the prototype attraction loss and the category disentanglement loss. The best results are highlighted in bold.

Datasets	$L_{\mathrm{cls}}$	$L_{\mathrm{cd}}$	Acc (%)	Precision (%)	Recall (%)	Macro-F1 (%)
Scene15			84.1	83.2	83.2	83.0
	✓		85.1	84.7	84.2	84.2
		✓	85.8	85.1	85.1	85.0
	✓	✓	**86.1**	**85.4**	**85.3**	**85.2**
CCV			58.6	55.8	56.0	55.7
	✓		59.0	56.2	56.4	56.0
		✓	59.1	56.1	56.4	56.1
	✓	✓	**59.7**	**57.0**	**56.9**	**56.6**

**Table 8:** Classification accuracy (%) comparison between MORG and traditional graph methods on Scene15 and CCV datasets. The best results are highlighted in bold.

Datasets	Graph-Free	FCG	*k*-NN			MORG (Ours)
			k=5	k=10	k=50	
Scene15	85.0	62.4	72.7	74.6	65.4	**86.1**
CCV	58.6	39.1	41.4	42.3	39.4	**59.7**

**Table 9:** Incremental ablation study on the Scene15 and CCV datasets. The best results are highlighted in bold.

Datasets	$L_{\mathrm{VIC}}$	$L_{\mathrm{IN}}$	$L_{\mathrm{CAT}}$	Acc (%)	Precision (%)	Recall (%)	Macro-F1 (%)
**Scene15**	✓			70.8	69.3	69.4	69.0
	✓	✓		84.1	83.2	83.2	83.0
	✓	✓	✓	**86.1**	**85.4**	**85.3**	**85.2**
**CCV**	✓			42.8	40.1	39.7	38.2
	✓	✓		58.6	55.8	56.0	55.7
	✓	✓	✓	**59.7**	**57.0**	**56.9**	**56.6**

**Table 10:** Macro-level ablation study of the tri-level constraints on the Scene15 and CCV datasets. The best results are highlighted in bold.

Datasets	$L_{\mathrm{VIC}}$	$L_{\mathrm{IN}}$	$L_{\mathrm{CAT}}$	Acc (%)	Precision (%)	Recall (%)	Macro-F1 (%)
Scene15		✓	✓	84.6	84.0	83.3	83.2
	✓		✓	84.0	83.8	82.9	82.9
	✓	✓		84.1	83.2	83.2	83.0
	✓	✓	✓	**86.1**	**85.4**	**85.3**	**85.2**
CCV		✓	✓	58.7	56.0	56.3	55.8
	✓		✓	57.5	55.2	55.3	55.0
	✓	✓		58.6	55.8	56.0	55.7
	✓	✓	✓	**59.7**	**57.0**	**56.9**	**56.6**

## Data Availability

The data presented in this study are available on request from the author.
